# Threatened Miscarriage in a COVID-19 Patient

**DOI:** 10.7759/cureus.31637

**Published:** 2022-11-18

**Authors:** Arshaan Momin, Natalie N Nguyen, Thor S Stead, Rohan K Mangal, Marcos Sosa, Kunal Patel, Latha Ganti

**Affiliations:** 1 Biology, Meridian World School, Round Rock, USA; 2 Biology and Medicine, Brown University, Providence, USA; 3 Medicine, The Warren Alpert Medical School of Brown University, Providence, USA; 4 Medicine, University of Miami Miller School of Medicine, Miami, USA; 5 Obstetrics and Gynecology, Lakeland Regional Health, Lakeland, USA; 6 Emergency Medicine, HCA Florida Ocala Hospital, Ocala, USA; 7 Emergency Medicine, Envision Physician Services, Plantation, USA; 8 Emergency Medicine, University of Central Florida College of Medicine, Orlando, USA

**Keywords:** imdevimab, casirivimab, unvaccinated covid-19, threatened miscarriage, covid-19

## Abstract

The authors present the case of a 30-year-old female who was pregnant and contracted COVID-19. She presented with vaginal bleeding and eventually went on to miscarry. Here, we discuss the risk factor of COVID-19 for threatened miscarriage and spontaneous abortion as well as pregnancy being a risk factor for increased COVID-19 disease severity. The patient received casirivimab and imdevimab per emergency department protocol due to this increased risk.

## Introduction

Analysis of data from the National Hospital Ambulatory Medical Care Survey from 1993 to 2003 showed that there are approximately 500,000 emergency department visits for vaginal bleeding in early pregnancy (VBEP) annually [1]. In the emergency department, VBEP is typically investigated through pelvic ultrasound or quantitative beta-human chorionic gonadotropin measurements [2]. The VBEP that occurs between six to eight weeks of gestation has been linked to an increased risk of clinical pregnancy loss [3]. Early pregnancy is a particularly vulnerable time, as 80% of spontaneous abortions occur within the first 12 weeks of pregnancy [4]. Since the advent of severe acute respiratory syndrome coronavirus 2 (SARS-CoV-2), also known as COVID-19, pregnant women have been alerted to their heightened susceptibility to infection. There is still little known about the effects of COVID-19 infections during pregnancy, but one retrospective cohort study in Canada found that the first-trimester miscarriage rate was unchanged from baseline in asymptomatic women [5]. However, a retrospective study in Washington state showed that nearly all pregnant women with COVID-19 infections were symptomatic [6], and maternal respiratory infection during pregnancy has been associated with a decrease in the gestational age of newborns [7]. Due to the ever-changing nature of the SARS-CoV-2 virus and the ongoing COVID-19 pandemic, clinicians should continue to monitor pregnant women with COVID-19 symptoms carefully until more research is done.

## Case presentation

A 30-year-old female G2P1001 without any medical history who was known to be COVID-19 positive and approximately six weeks pregnant presented to our emergency department due to vaginal bleeding. She was worried about a miscarriage. Her vaginal bleeding began the previous evening and was persistent on the morning of the emergency department (ED) visit. She denied any fevers, chills, nausea, vomiting, diarrhea, or dysuria. She reported mild pelvic cramping. Besides being COVID-19 positive, she denied any past medical history, past surgical history, or any medication allergies. She was not vaccinated against COVID-19.

Vital signs in the emergency department revealed a temperature of 98° F, a pulse of 99 beats per minute, respiration of 18 breaths per minute, blood pressure of 124/90 mmHg, and saturation at 100% on room air. Physical examination was unremarkable except for vaginal bleeding. The cervical os was closed. 

**Figure 1:**
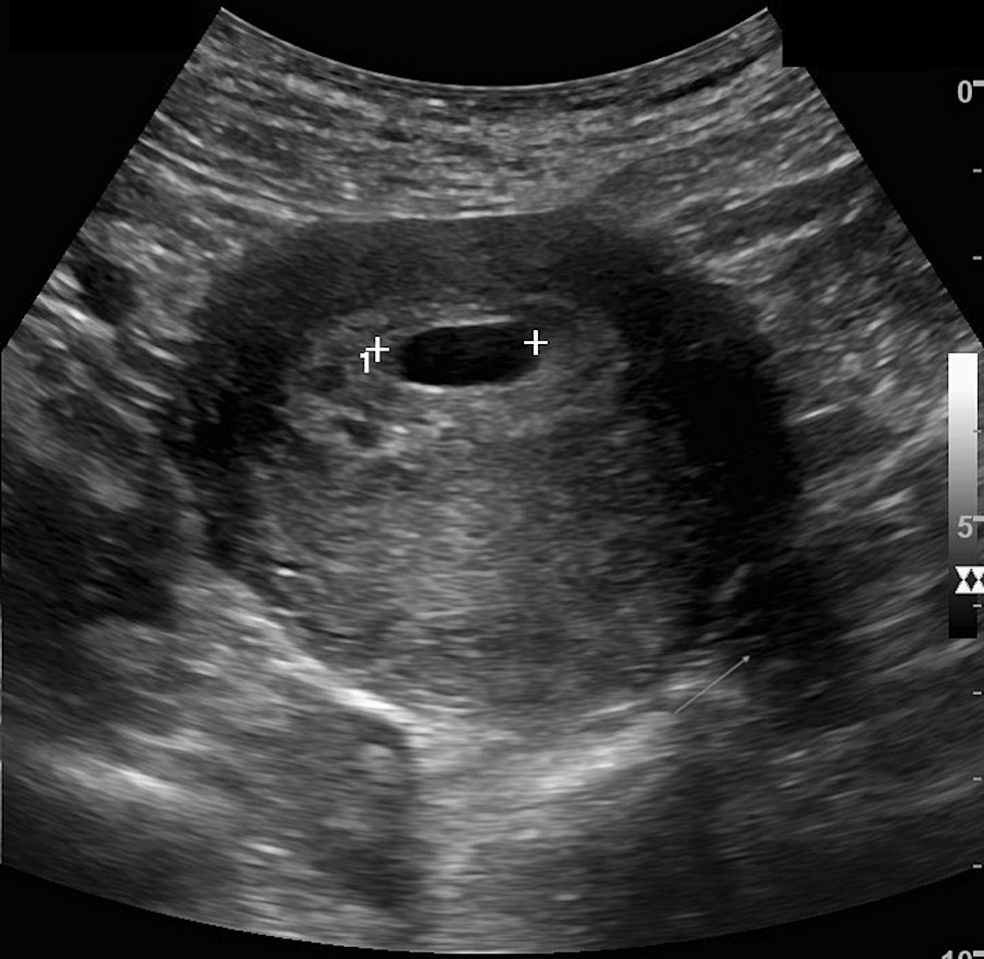
Transverse view of the uterus demonstrating gestational sac (white crosshatch) and small subchorionic hemorrhage (arrow). No fetal pole or yolk sac was seen.

Laboratory analysis was significant for a beta-human chorionic gonadotropins (hCG) level of 13,483. Transvaginal ultrasonography revealed an intrauterine gestational sac with measurements corresponding to approximately five weeks and six days. No fetal pole or yolk sac was identified. A small subchorionic hemorrhage was noted (Figure 1).

Given that pregnancy was an added risk factor for disease progression and COVID-19, the patient was offered and chose to receive monoclonal antibodies (mAb). She received casirivimab and imdevimab as per emergency department protocol. The patient tolerated the infusion well without any complications. She was discharged from the emergency department with strict instructions to return in two days for repeat beta-hCG testing. 

On her return visit, i.e., two days after the initial visit, the patient remained COVID-19 positive and continued to bleed although the quantity and briskness were not worsened. The repeat beta-hCG level was 14,566. The patient was discharged from the emergency department with obstetrics and gynecology follow-up and threatened miscarriage precautions. The patient followed up with her obstetrician/gynecologist (OBGYN) as instructed, and a follow-up sonogram confirmed a completed miscarriage.

## Discussion

Though pregnant women are considered a high-risk group for COVID-19 infection, particularly those with comorbidities such as obesity or asthma [6], an early report published by the World Health Organization in 2020 showed that of 147 pregnant women with COVID-19 infections, 8% were categorized as severely ill and 1% were categorized as critically ill [8], compared to the 14% and 5% of the general population COVID-19 infections that were categorized as severe and critical, respectively [9,10]. Bukowska-Ośko et al. have hypothesized that the antiviral effects of lactoferrin may inhibit SARS-CoV-2 infection [11]. It is unclear whether being pregnant may escalate the severity of a concurrent SARS-CoV-2 infection, but some research has shown that a SARS-CoV-2 infection is associated with severe maternal death and morbidity from obstetric complications, such as hypertension, postpartum hemorrhage, or other infection [12]. There is not yet any research published that has found an association between SARS-CoV-2 infection and vaginal bleeding; however, an observational cohort study in the USA found 63,815 females had irregular menses or vaginal bleeding after receiving the COVID-19 vaccine [13].

There are also not yet any conclusive studies published specifically about the SARS-CoV-2 effect on early pregnancy, so researchers have looked at the SARS effect on early pregnancy to extrapolate the SARS-CoV-2 effect on early pregnancy, as SARS-CoV-2 shares 80% genome similarity with the SARS virus [14,15]. It has been reported that four of seven women with SARS infection in the first trimester of pregnancy had spontaneous abortions [15,16]. However, it is notable that SARS-CoV-2 has a much lower case fatality rate than SARS and appears to have less severe maternal pregnancy outcomes than SARS [17,18]. Therefore, it may be concluded that while SARS-CoV-2 infection in early pregnancy may increase the risk of spontaneous abortion, its effect will be less severe than that of SARS. 

One United Kingdom study of 3545 women in their first trimester of pregnancy reported a 14% rate of early miscarriage in the COVID-19 infected group and 8% in the uninfected group. This finding retained statistical significance after adjusting for age, body mass index (BMI), ethnicity, smoking status, gestational age, and the number of previous miscarriages (relative rate 1.7, 95% confidence interval (CI) 1.0-3.0, P=0.06) [19]. Awaited are the results of a systematic review protocol examining COVID-19 during pregnancy and the risk of pregnancy loss that has been registered with the International Prospective Register of Systematic Reviews (PROSPERO) [20]. 

If the SARS-CoV-2 infection occurs in the second or third trimesters, it can lead to poor birth outcomes. No reports of SARS-CoV-2 transmission from mother to child during the first trimester of pregnancy have been reported, but there are reports of rare cases of viral transmission to the fetus through the placenta in the second and third trimesters [15]. Vertical transmission of the virus occurs through the placental tissue, as the placenta can be infected by SARS-CoV-2 [15,18]. Infection of the placenta can result in SARS-CoV-2 placentitis, which can then lead to malperfusion, placental insufficiency, and ultimately, stillbirth or neonatal death [18]. Around 47% of women who were hospitalized for COVID-19 had preterm deliveries, however, of the neonates born to mothers with SARS-CoV-2 infections, 95% were healthy [21-23].

To improve maternal and neonatal outcomes, pregnant women are able to undergo various treatments for SARS-CoV-2 infection. As COVID-19 is a risk factor for venous thromboembolism, prophylactic-dose anticoagulation is recommended for severe infections that require hospitalization [17]. Steroids, such as dexamethasone, are also recommended in severe cases that require supplemental oxygen or ventilation. Additionally, dexamethasone has a positive effect on fetal lung maturity in preterm births [17]. Other possible interventions recommended by the National Institutes of Health (NIH) include antiviral treatments, such as ritonavir-boosted nirmatrelvir and remdesivir, or mAb when antiviral treatments are not possible or appropriate [21]. Monoclonal antibody therapy has only recently been approved for emergency COVID-19 treatment, so there is not much research done on the effects of antenatal anti-SARS-CoV-2 mAb exposure. Though there is no evidence of adverse pregnancy outcomes, such as miscarriage, preterm delivery, or congenital malformations, it is known that most mAb are able to cross the placenta easily, so infants who were exposed to antenatal mAb should be monitored carefully, particularly after treatment with any newer anti-SARS-CoV-2 mAb [24-26].

## Conclusions

In the clinical case described in this paper, the patient was Covid-positive and approximately five to six weeks pregnant. She initially presented to the emergency department for vaginal bleeding and received a course of mAb at the time. However, the patient returned two days later with the diagnosis of threatened abortion, which contributes to the hypothesis that COVID-19 infection during early pregnancy likely increases the risk of spontaneous abortion. This case is also unique in that the VBEP may be linked to the maternal SARS-CoV-2 infection. Furthermore, the effects of anti-SARS-CoV-2 mAb treatment in early pregnancy have not been well documented. Due to the gaps in knowledge of COVID-19 and pregnancy, clinical cases with unique features, such as this one, should be well documented and studied as the pandemic continues.

## References

[1] Wittels KA, Pelletier AJ, Brown DF, Camargo CA Jr (2008). United States emergency department visits for vaginal bleeding during early pregnancy, 1993-2003. Am J Obstet Gynecol.

[2] McKennett M, Fullerton JT (1995). Vaginal bleeding in pregnancy. Am Fam Physician.

[3] Hossain R, Harris T, Lohsoonthorn V, Williams MA (2007). Risk of preterm delivery in relation to vaginal bleeding in early pregnancy. Eur J Obstet Gynecol Reprod Biol.

[4] Everett C (1997). Incidence and outcome of bleeding before the 20th week of pregnancy: prospective study from general practice. BMJ.

[5] Rotshenker-Olshinka K, Volodarsky-Perel A, Steiner N, Rubenfeld E, Dahan MH (2021). COVID-19 pandemic effect on early pregnancy: are miscarriage rates altered, in asymptomatic women?. Arch Gynecol Obstet.

[6] Lokken EM, Walker CL, Delaney S (2020). Clinical characteristics of 46 pregnant women with a severe acute respiratory syndrome coronavirus 2 infection in Washington State. Am J Obstet Gynecol.

[7] Yang J, Zheng Y, Gou X (2020). Prevalence of comorbidities and its effects in patients infected with SARS-CoV-2: a systematic review and meta-analysis. Int J Infect Dis.

[8] Guo LQ, Zhao DD, Liu R (2018). A propensity score-matched study on relationship between maternal respiratory infection in early pregnancy and gestational age. Zhonghua Liu Xing Bing Xue Za Zhi.

[9] (2022). Report of the WHO-China Joint Mission on Coronavirus Disease 2019 (COVID-19). https://www.who.int/publications/i/item/report-of-the-who-china-joint-mission-on-coronavirus-disease-2019-(covid-19).

[10] Wu Z, McGoogan JM (2020). Characteristics of and important lessons from the coronavirus disease 2019 (COVID-19) outbreak in China: summary of a report of 72314 cases from the Chinese Center for Disease Control and Prevention. JAMA.

[11] Bukowska-Ośko I, Popiel M, Kowalczyk P (2021). The immunological role of the placenta in SARS-CoV-2 infection-viral transmission, immune regulation, and lactoferrin activity. Int J Mol Sci.

[12] Metz TD, Clifton RG, Hughes BL (2022). Association of SARS-CoV-2 infection with serious maternal morbidity and mortality from obstetric complications. JAMA.

[13] Wong KK, Heilig CM, Hause A (2022). Menstrual irregularities and vaginal bleeding after COVID-19 vaccination reported to v-safe active surveillance, USA in December, 2020-January, 2022: an observational cohort study. Lancet Digit Health.

[14] Mullins E, Evans D, Viner RM, O'Brien P, Morris E (2020). Coronavirus in pregnancy and delivery: rapid review. Ultrasound Obstet Gynecol.

[15] Lu R, Zhao X, Li J (2020). Genomic characterisation and epidemiology of 2019 novel coronavirus: implications for virus origins and receptor binding. Lancet.

[16] Wang CL, Liu YY, Wu CH, Wang CY, Wang CH, Long CY (2021). Impact of COVID-19 on pregnancy. Int J Med Sci.

[17] Wong SF, Chow KM, Leung TN (2004). Pregnancy and perinatal outcomes of women with severe acute respiratory syndrome. Am J Obstet Gynecol.

[18] Berlutti F, Pantanella F, Natalizi T, Frioni A, Paesano R, Polimeni A, Valenti P (2011). Antiviral properties of lactoferrin—a natural immunity molecule. Molecules.

[19] Balachandren N, Davies MC, Hall JA (2022). SARS-CoV-2 infection in the first trimester and the risk of early miscarriage: a UK population-based prospective cohort study of 3041 pregnancies conceived during the pandemic. Hum Reprod.

[20] Campbell J, Williams R, Harley M, Bhaskaran K (2022). COVID-19 during pregnancy and risk of pregnancy loss (miscarriage or stillbirth): a systematic review protocol. BMJ Open.

[21] Schwartz DA (2022). Stillbirth after COVID-19 in unvaccinated mothers can result from SARS-CoV-2 pacentitis, placental insufficiency, and hypoxic ischemic fetal demise, not direct fetal infection: potential role of maternal vaccination in pregnancy. Viruses.

[22] Pham-Huy A, Top KA, Constantinescu C, Seow CH, El-Chaâr D (2021). The use and impact of monoclonal antibody biologics during pregnancy. CMAJ.

[23] Şahin D, Tanaçan A, Webster SN, Moraloğlu Tekin Ö (2021). Pregnancy and COVID-19: prevention, vaccination, therapy, and beyond. Turk J Med Sci.

[24] Allotey J, Stallings E, Bonet M (2020). Clinical manifestations, risk factors, and maternal and perinatal outcomes of coronavirus disease 2019 in pregnancy: living systematic review and meta-analysis. BMJ.

[25] (2022). COVID-19 Treatment Guidelines. https://www.covid19treatmentguidelines.nih.gov.

[26] Pham-Huy A, Sadarangani M, Huang V (2019). From mother to baby: antenatal exposure to monoclonal antibody biologics. Expert Rev Clin Immunol.

